# *Plasmodium falciparum* DHFR and DHPS Mutations Are Associated With HIV-1 Co-Infection and a Novel DHPS Mutation I504T Is Identified in Western Kenya

**DOI:** 10.3389/fcimb.2020.600112

**Published:** 2020-11-26

**Authors:** Brandi K. Torrevillas, Sarah M. Garrison, Alexander J. McKeeken, Dharmeshkumar Patel, James T. Van Leuven, Nathaniel I. Dizon, Karina I. Rivas, Nicholas J. Hathaway, Jeffrey A. Bailey, John N. Waitumbi, Carolyne M. Kifude, Janet Oyieko, V. Ann Stewart, Shirley Luckhart

**Affiliations:** ^1^Department of Entomology, Plant Pathology and Nematology, University of Idaho, Moscow, ID, United States; ^2^Institute for Modeling Collaboration and Innovation, University of Idaho, Moscow, ID, United States; ^3^Department of Biological Sciences, University of Idaho, Moscow, ID, United States; ^4^Department of Preventive Medicine and Biostatistics, Uniformed Services University of the Health Sciences, Bethesda, MD, United States; ^5^Program in Bioinformatics and Integrative Biology, University of Massachusetts, Worcester, MA, United States; ^6^Department of Pathology and Laboratory Medicine, Brown University, Providence, RI, United States; ^7^Basic Science Laboratory, United States Army Medical Research Directorate-Africa, Kenya Medical Research Institute, Kisumu, Kenya

**Keywords:** antimalarial drug resistance, antifolate therapy, HIV-1, asymptomatic malaria, targeted deep sequencing, complexity of infection, *Plasmodium falciparum*

## Abstract

Antifolate resistance is significant in Kenya and presumed to result from extensive use and cross-resistance between antifolate antimalarials and antibiotics, including cotrimoxazole/Bactrim used for HIV-1 chemotherapy. However, little is known about antifolate-resistant malaria in the context of newly diagnosed HIV-1 co-infection prior to administration of HIV-1 chemotherapy. Blood samples from a cross-sectional study of asymptomatic adult Kenyans enrolled during voluntary HIV testing were analyzed by PCR for *Plasmodium* spp. More than 95% of volunteers with identifiable parasite species (132 HIV-1 co-infected) were infected with *Plasmodium falciparum* alone or *P. falciparum* with *Plasmodium ovale* and/or *Plasmodium malariae*. Deep sequencing was used to screen for mutations in *P. falciparum dihydrofolate reductase (dhfr)* (N51I, C59R, S108N, I164L) and *dihydropteroate synthase (dhps)* (S436H, A437G, K540E, A581G) from 1133 volunteers. Individual mutations in DHPS but not DHFR correlated with HIV-1 status. DHFR haplotype diversity was significantly different among volunteers by gender and HIV-1 status. DHPS haplotype diversity by HIV-1 status was significantly different between volunteers paired by age and gender, indicating that patterns of resistance were independent of these variables. Molecular simulations for a novel DHPS mutation (I504T) suggested that the mutated protein has increased affinity for the endogenous ligand DHPPP and decreased affinity for drug binding. A sub-group of monoclonal infections revealed that age and parasitemia were not correlated and enabled identification of a rare septuple-mutant haplotype (IRNL-HGEA). In our study, adult Kenyans newly diagnosed with HIV-1 infection were predominantly infected with moderately resistant *P. falciparum*, with patterns of infecting parasite genotypes significantly associated with HIV-1 status. Together with the discovery of DHPS I504T, these data indicate that antifolate resistance continues to evolve in Kenya. Further, they highlight the need to understand the effects of associated mutations on both fitness and resistance of *P. falciparum* in the context of HIV-1 co-infection to better inform treatment for asymptomatic malaria.

## Introduction

Repeated episodes of malaria typically result in partial protective immunity in regions of stable and holoendemic transmission. In this context, partial immunity can facilitate chronic parasite carriage and asymptomatic infection, challenging efforts to reduce transmission. Such persistent, sub-patent infections can also sustain parasite genetic diversity, including those genotypes that are drug-resistant, as the predominant reservoir for parasite transmission (Tadesse et al., 2018). For example, a higher prevalence of drug-resistant parasites was detected in a 2014 study of asymptomatic parasitemic Ugandan children relative to infected children with fever (Tukwasibwe et al., 2014). In a broader context, the number of parasite clones infecting a single host, defined as complexity of infection (COI), has been correlated with enhanced protection against subsequent clinical malaria (Sondén et al., 2015), but elevated COI has also been suggested to favor survival and transmission of the infecting parasites (Wargo et al., 2007). Accordingly, the presence of multiple parasite lineages within a single host can drive the spread of drug resistance, as has been shown experimentally in a mouse malaria model following drug administration (de Roode et al., 2004). The interactions among host immunity, antimalarial drug resistance, disease severity and risk of parasite transmission are further complicated in the presence of HIV-1. Notably, the prevalence of asymptomatic malaria has been variously reported in children and adults to be higher or lower in the context of HIV-1 co-infection (Missinou et al., 2003) (reviewed in Steketee et al., 1996; Kalyesubula et al., 1997; Whitworth et al., 2000; Flateau et al., 2011), highlighting the difficulty in discerning patterns across such studies. Of particular relevance for our studies, Rutto et al. noted higher mean parasite densities in HIV-1 co-infected individuals relative to HIV-1 negative individuals in western Kenya (Rutto et al., 2015).

Early concerns regarding HIV-1 co-infection prompted studies that suggested mutations in parasite dihydrofolate reductase (*dhfr)* and dihydropteroate synthase (*dhps)* due to use of trimethoprim-sulfamethoxazole (Bactrim), a commonly prescribed preventative for HIV-associated opportunistic infections, provided cross resistance to the antimalarial sulfadoxine-pyrimethamine (SP) (Iyer et al., 2001). While SP is no longer a first line antimalarial, this combination is still recommended for intermittent preventative treatment in pregnancy and in children in combination with amodiaquine for seasonal malaria chemoprevention in endemic countries, including Kenya. In a recent paper, Juma et al. (Juma et al., 2019) reported the unexpected findings that cessation of Bactrim prophylaxis was associated with significantly increased, rather than reduced frequency of SP resistance mutations in *P. falciparum* DHFR (N51I, C59R, S108N) and DHPS (A437G, K540E) in HIV-infected volunteers in western Kenya. The authors concluded that Bactrim lowers the incidence of SP-resistant parasites (Juma et al., 2019), suggesting that prolonged use of Bactrim for HIV-1 supportive therapy can limit both parasite infection and antimalarial resistance, and that in the absence of Bactrim, SP resistance mutations confer a fitness advantage over SP-susceptible genotypes. However, Juma et al. acknowledged the lack of an HIV-negative control group as a limitation for interpretation of their data.

In light of existing work and unanswered questions regarding associations among COI, antifolate resistance, and HIV-1 co-infection in malaria, we used deep sequencing to analyze *P. falciparum dhfr* and *dhps* mutations, as well as three loci in highly polymorphic genes whose allelic diversity is often used to estimate COI. We amplified these targets from blood samples from a cross-sectional study of Kenyan adults who self-presented for voluntary HIV testing and were, therefore, not currently on any HIV chemotherapeutic regimen. While our findings did not reveal significant associations of COI with HIV-1 status, we did identify HIV-associated differences in the prevalence of encoded DHFR and DHPS mutations and haplotypes that were age- and gender-dependent, suggesting that HIV-1 co-infection is associated with altered drug resistance in *P. falciparum* in the absence of HIV chemotherapy. We also identified I504T, a new mutation in DHPS, that is predicted to enhance binding of the endogenous substrate dihydropterin pyrophosphate (DHPPP) and to function cooperatively with A437G and K540E to reduce drug binding. Collectively, these findings indicate that antifolate resistance is continuing to evolve in Kenya and highlight the need to better understand the effects of associated mutations on both fitness and resistance in *P. falciparum*.

## Methods

### Sample Collection and Genomic DNA Extraction

Asymptomatic adults (≥ 18 years old) seeking voluntary HIV testing and counseling at the HIV Testing and Counseling (HTC) Center, Kisumu West Hospital, Kombewa, Nyanza Province, Kenya, or an associated HTC Center in the Kisumu West District (within the Kisumu West District WRP/KEMRI PEPFAR program) between January of 2015 and July of 2018 were asked to participate in a malaria study under guidance by the Institutional Review Boards of the Uniformed Services University of the Health Sciences (USUHS# G18753), Walter Reed Army Institute of Research (WRAIR #2033) and the Kenya Medical Research Institute (KEMRI, protocol #2600; Janet Oyieko, Clinical Principle Investigator). Self-report of pregnancy was an exclusion criterion. A total of 1762 individuals were enrolled. 1133 yielded detectable signal from blood samples in a quantitative PCR (qPCR) assay for the *Plasmodium* genus-specific *18S ribosomal RNA* gene and 132 were HIV-1 co-infected; among volunteers for which infection could be determined to species (n=670), more than 90% were positive for *P. falciparum* infection only (n=606), while an additional 6.0% were positive for infection with *P. falciparum* in combination with *P. malariae* (n= 28) or *P. ovale* (n=9), or infected with all three (n=3) (C. Kifude, pers. comm). Dried blood spots prepared at the time of enrollment were shipped from study sites to USUHS for nucleic acid isolation on the QIACube automated platform (QIAGEN, Hilden, Germany). Purified genomic DNA samples were then shipped to the University of Idaho for targeted deep sequencing of *P. falciparum* genes of interest.

### Nested Dual-Barcoded PCR Library Preparation and Illumina Sequencing

Because many volunteers had low *18S* copy numbers, all genomic DNA samples were amplified by primary PCR in duplicate to enrich each of the five *P. falciparum* target loci subsequently used as templates for two-step, dual-barcoded library sequencing designed and optimized by the University of Idaho Genomic Resources Core (SOP#GRC_008). Primer sequences are included in **Supplemental Table 1** (Duraisingh et al., 1998; Ye et al., 2012; Taylor et al., 2013). Specifically, we amplified sequences of interest from genomic DNA using KAPA HiFi HotStart polymerase (Kapa Biosystems, Wilmington, MA, USA) for an initial 30 cycles according to manufacturer’s instructions. Samples were included in subsequent steps if the initial amplification of *dhps* produced an appropriately-sized visible band (a qualitative predictor of deep-sequencing success) following electrophoresis through 2% agarose with ethidium bromide (70V for 2 h). The *dhps*-positive samples were subjected to amplification of mutation-containing regions of *dhfr* and *dhps* and for three highly polymorphic loci for determination of COI, including regions of the *circumsporozoite protein (csp)* gene and the *apical membrane antigen 1* gene domains 1 and 2 (*ama d1* and *ama1 d2*). Primary amplicons were nested with target-specific primers bearing universal common sequence (CS) tags (Fluidigm Corporation, South San Francisco, CA, USA) complementary to barcode primers used downstream to label pooled targets. Tagged amplimers were quantified using Nanodrop ND-1000 (Thermo Fisher Scientific, Waltham, MA, USA). Amplimers were normalized in molecular biology grade water and pooled in equimolar ratios to a final concentration of 100 ng/µl. Pooled targets (two pools per volunteer, one per PCR replicate) were cleaned using ChargeSwitch magnetic beads (Invitrogen, Carlsbad, CA, USA) on the KingFisher Flex automated plate-based extraction instrument (Thermo Fisher Scientific, Waltham, MA, USA) using a custom script provided by Thermofisher technical support.

Cleaned amplimer pools for each sample replicate were used as input template for dual-barcoding PCR to label targets within each replicate pool with a unique sample index. Custom barcoding primer pairs contained sequences complementary to Fluidigm CS-tags, followed by an 8bp unique sequence barcode and Illumina P5 or P7 primer to generate a sequenceable library. Barcoded libraries were combined by qualitative agarose gel score (two bands, one band, no band). Combined libraries were cleaned at the University of Idaho IBEST Genomic Resources Core by size selection *via* Ampure bead cleanup (Beckman Coulter, IN, USA) keeping all fragments greater than 450 base pairs. Cleaned, size-selected libraries were examined with a Fragment Analyzer (Agilent, Santa Clara, CA, USA) to verify library quality and composition. Each library was quantified by qPCR (Kapa Biosystems, Wilmington, MA, USA), then pooled to equimolar ratios and sequenced on a quarter lane of Illumina MiSeq 2x300 (Illumina, Inc. San Diego, CA, USA) producing ~5 million reads demultiplexed by barcode and target-specific primers using dbcAmplicons (Settles, 2019).

The library prep protocol was validated using genomic DNA from *P. falciparum* strains V1/S (MRA-176, BEI Resources, Manassas, VA) and D10-sgkga (MRA-559) with known mutations. *dhfr* and *dhps* were amplified from these strains and mixed with amplimers from strain NF54 e2 (MRA-1000) in molar ratios of 1%, 5%, 95%, and 99%. Mixed amplimers were used as primary PCR products and subjected to adapter and barcode PCR as above, then analyzed on a full lane of Illumina MiSeq 2x300 to confirm detection of expected mutations (data not shown).

### Sequence Data Analyses and Statistics

Raw sequence reads were parsed using the Python script split_library.py in the QIIME bioinformatics pipeline (Caporaso et al., 2010). The SeekDeep pipeline (v. 3.0.1-dev) (Hathaway et al., 2018) for targeted amplicon analysis was used with default parameters for Illumina sequencing initiated with the utility command setupTarAmpAnalysis which creates a tree of sample directories to pass sequence data into processes for analysis, generate folders for output, and create scripts for the pipeline. The analysis was performed with the script runAnalysis.sh and results were ported into a browser for visualization using the script startServerCmd.sh. During haplotype clustering, duplicate PCR libraries were used to screen out stochastic amplification errors and chimeric false haplotypes. Mutations were confirmed only when present in both PCR replicates with haplotype frequency at least 1% and greater than 100 reads. Based on homology analysis, all amplicons from the five target genes were determined to derive from *P. falciparum* genomic DNA amplification. Haplotypes output by SeekDeep were encoded to 4-letter abbreviations based on canonical SNPs in DHFR (N51I, C59R, S108N, and I164L) and DHPS (S436A/H, A437G, K540E, and A581G).

Parasite sequence data for *dhfr* (n=332) and *dhps* (n=417) were included for analyses of mutations and haplotypes using the following two approaches and Chi-square analysis. First, we analyzed proportions of encoded DHFR and DHPS mutations among HIV-positive versus HIV-negative volunteers. Second, we analyzed proportions of haplotypes among volunteers paired by HIV-1 status, gender and age. Haplotypes were assessed as proportion of haplotypes by group, with groups defined by HIV-1 status and gender. Each haplotype was regarded as one parasite type in the parasite population across all volunteers rather than haplotype by volunteer to simplify the analysis and control for COI >1. Each volunteer with at least one haplotype determined from *ama1* or *csp* was given a COI score based on the number of unique alleles returned for each target. The highest number of alleles returned for any of the COI targets was considered to be the most likely number of clones carried by that individual. Volunteers with sequence data from at least one COI target (n=565) were analyzed by Spearman’s Rho nonparametric test for correlation between parasitemia and COI and between age and COI. Pairwise t-tests for COI were conducted for volunteers grouped by gender and HIV-1 status. Spearman’s Rho nonparametric test for correlation and t-tests were performed on GraphPad Prism 8.3.0 (GraphPad Software, San Diego, CA USA). Chi-square analyses were performed using library package Rcompanion on RStudio (RStudio, Inc., Boston, MA). All analyses were conducted at 95% confidence (α=0.05).

For age- and gender-matched volunteer pair analyses, all parasite haplotypes (*dhfr* n=332, *dhps* n=417) were sorted by host volunteer HIV-1 status, then by volunteer gender and finally by volunteer age. Haplotypes from volunteers with inconclusive HIV-1 status—five DHFR haplotypes from 4 volunteers and three DHPS haplotypes from two volunteers—were excluded from the analysis. For every HIV-positive volunteer, one volunteer of the same gender and age was selected at random, with *18S* copy numbers blinded to avoid bias. Individuals over age 40 comprised only 10% (111/1133) of volunteers and were infrequent among volunteers with data for *dhfr* (20/320 volunteers or 6.25%) and for *dhps* (22/360 volunteers or 6.1%). For *dhfr*, all matched pairs were within two years’ difference in age except for the oldest matched pairs which had an age difference of four years (males) and seven years (females). For *dhps*, all matched pairs were within two years’ difference in age.

As an external control to validate the stringency of filtering parameters utilized by SeekDeep, raw sequence reads were also analyzed by the Genome Analysis Toolkit (GATK) (McKenna et al., 2010) variant analysis program HaplotypeCaller in the Variant Call Format (VCF) Toolkit (Danecek et al., 2011). Reads were adapter-trimmed and demultiplexed using dbcAmplicons then and quality filtered with fastp v0.19.6 (Chen et al., 2018) using default parameters. Forward and reverse reads were mapped to *dhps* (U07706.1), *dhfr* (EU046230.1), *ama1* (XM_001347979.1), and *csp* (XM_001351086) reference sequences using Burroughs-Wheeler Aligner (BWA) (Li and Durbin, 2009). Read counts per sample output by BWA-mem were parsed using Sequence Alignment/Map tools (Li et al., 2009) to explore the global number of reads mapping to any locus. This treatment identified disparities between the numbers of reads kept by mapping using the BWA-mem compared to those discarded by SeekDeep for failing overlap quality filtering. To identify SNPs in our data, reads were processed using SAMtools followed by the VCF Toolkit (Danecek et al., 2011) to produce mpileup, flagstat, and vcf files. GATK v3.8 (McKenna et al., 2010) was used to combine individual vcf files from which variants were identified. In addition to default filtering performed by vcfutils.pl varFilter, we imposed a minimum 100x coverage cutoff. R was used to parse information from these files, enabling validation of SNP calls and frequencies identified by SeekDeep.

Combined output files from SeekDeep were parsed in R and compared to the SNPs identified with VCFtools. Identical haplotypes from different SeekDeep runs were merged using the CD-HIT (cd-hit-est) web server (Huang et al., 2010). After merging SeekDeep runs, we removed contaminating human amplicons from *ama1 d1* data by aligning haplotypes to *ama1* reference sequence, then using blastn against non-redundant sequences to identify the source of extremely divergent sequences. Phangorn (Schliep, 2011), ape (Paradis and Schliep, 2019), and ade4 (Dray and Dufour, 2007) were used in R to perform sequence alignments, model testing, and to build Neighbor-Joining trees. No indels were observed in the final SeekDeep haplotypes. For *ama1* and *csp*, the within-host frequencies of malaria parasite haplotypes were plotted with the phylogenetic tree of haplotypes using ggtree v2.0.0 (Yu et al., 2017).

### Molecular Modeling of *P. falciparum* DHPS

The 3D structure of *P. falciparum* DHPS was not available at the time this study was initiated but has recently become available (Chitnumsub et al., 2019). Accordingly, we used a crystal structure of *P. vivax* DHPS in complex with substrates 6-hydroxymethyl-7,8-dihydropterin pyrophosphate (DHPPP) and 4-aminobenzoic acid (*p*ABA) (Yogavel et al., 2018) to build a homology model of *P. falciparum* DHPS, which we subsequently compared to the published structure (Chitnumsub et al., 2019). The experimental structure of *P. vivax* DHPS in complex with DHPPP and *p*ABA substrates was downloaded from the Protein Data Bank (PDB) server (PDB ID: 5Z79) (Manickam et al., 2018) and the structure prediction wizard from the PRIME module of Schrodinger suite was used for homology modeling (Jacobson et al., 2002; Jacobson et al., 2004). The substrates and Mg^+2^ ion were left in their respective binding sites in the homology modeling process since residues in the binding site are conserved between *P. falciparum* and *P. vivax* DHPS. Loops that were not in the template were refined using the loop refinement module of Schrodinger suite. Mutations in *P. falciparum* DHPS (A437G, I504T, K540E, and A437G+I504T+K540E) were generated using the “mutate residue” option in Maestro from Schrodinger suite and appropriate sidechain rotamers were selected based on the most probable rotamer list in Maestro (Schrödinger Release, 2020).

The homology model of *P. falciparum* DHPS and mutants were subjected to molecular dynamics (MD) simulations using GROMACS 2018.3 (Abraham et al., 2015) with the CHARMM36 forcefield (Huang and MacKerrel, 2013). Forcefield parameters of DHPPP and *p*ABA substrates were generated using the SwissParam webserver (Zoete et al., 2011) to understand the effects of DHPS mutations on drug binding, not to calculate absolute binding affinities to DHPPP and *p*ABA. The protein structures were solvated using TIP3P water molecules and neutralized using 0.15M Na^+^ and Cl^-^ ions. Each system was then minimized and equilibrated using harmonic restraints on the backbone of the protein and the heavy atoms of the substrates. Equilibration simulations were performed for 1 ns each using fixed NVT followed by fixed NPT conditions. Production simulations of each system were then performed for 100 ns using fixed NPT conditions with Parrinello-Rahman pressure coupling at 1 atm and v-rescale temperature coupling at 300 K. The time step was 2 fs and snapshots were saved at every 10 ps.

## Results

### COI and Parasitemia Were Predicted to Minimally Impact Patterns of DHFR and DHPS Mutations in Our Volunteers

Interpretable sequence data for DHFR (N51I, C59R, S108N, I164L) and DHPS (S436A/H, A437G, K540E, A581G) mutations were collected from 680 volunteers yielding 749 haplotype sequences, including 332 haplotype sequences for *dhfr* (from 320 volunteers, 36 HIV-1 co-infected) and 417 haplotype sequences for *dhps* (from 360 volunteers, 42 HIV-1 co-infected; **Table 1**). All haplotype sequences called by SeekDeep have been deposited in GenBank to be made publicly available upon publication of this manuscript (Luckhart et al., 2020). Overall, the VCF Toolkit (Danecek et al., 2011) called slightly fewer *dhps* SNPs and slightly more *dhfr* SNPs compared with SeekDeep (**Supplemental Figure 1**). The novel DHPS I504T mutation was detected by SeekDeep in both replicates for a single volunteer. This SNP was at ~6% frequency in the BWA alignment of reads but was not called by VCFtools (**Supplemental Figure 1**) due to its rarity within the total population after quality filtering and stitching by the VCF Toolkit (default parameters).

**Table 1:** Frequency of DHFR or DHPS haplotype.

Number of mutations*	0	1	2	3	4
**DHFR Haplotype** **(n=332 haplotype sequences)**	NCSI	NC**N**I	**I**C**N**I	**IRN**I **I**C**NL**	**IRNL**
Malaria-only volunteers (n=292 haplotype sequences)	3	0	9	273 4	3
HIV-1 co-infected volunteers (n=40 haplotype sequences)	0	0	3	34 1	2
**DHPS Haplotype** **(n=417 haplotype sequences)**	**SAKA**	S**G**KA **A**AKA	S**GE**A S**G**K**G**	S**GEG** **HGE**A S**GE**A+I504**T**	**HGEG**
Malaria-only volunteers (n=360 haplotype sequences)	3	5 2	246 2	3 97 1	1
HIV-1 co-infected volunteers (n=57 haplotype sequences)	0	1 0	36 0	0 20 0	0

*DHFR haplotypes included nonsynonymous mutations N51**I**, C59**R**, S108**N**, and I164**L**; DHPS haplotypes included S346**A/H**, A437**G**, K540**E**, and A581**G** and the novel mutation I504**T**. Malaria parasite infection was determined by qPCR of Plasmodium genus-specific 18S ribosomal RNA gene and HIV-1 status was confirmed by rapid diagnostic test (C. Kifude, pers. comm.).

While there were no differences in parasitemia (*18S* copies per µl) by age, HIV-1 status or gender, we stratified our sequence data for analysis not only by HIV-1 status but also by age and gender because these parameters were significantly different among our volunteers (C. Kifude, pers. comm.). Of 565 volunteers with sequence data from one or more COI targets, 305 volunteers had COI=1, 127 had COI=2, 133 had COI=3 or more, which translated to 76% of volunteers having one or two parasite clones (overall mean COI=1.94; **Table 2**).

**Table 2:** Distribution of loci sequenced for complexity of infection (COI).

COI (number of volunteers)	One target	number of volunteers in COI class	Two targets		Three targets
COI = 1 (n=305)	AMA1 d1 only: AMA1 d2 only: CSP only:	52 37 115	AMA1 d1 and AMA1 d2: AMA1 d1 and CSP: AMA1 d2 and CSP:	18 22 23	38
COI = 2 (n=127)	AMA1 d1 only: AMA1 d2 only: CSP only:	21 9 19	AMA1 d1 and AMA1 d2: AMA1 d1 and CSP: AMA1 d2 and CSP:	8 19 14	37
COI > 2 (n=133)	AMA1 d1 only: AMA1 d2 only: CSP only:	20 5 16	AMA1 d1 and AMA1 d2: AMA1 d1 and CSP: AMA1 d2 and CSP:	14 23 5	50
Mean COIs*		1.50		2.10	2.80

*Overall mean COI from all volunteers with any number of loci = 1.94.

**Figure 1:**
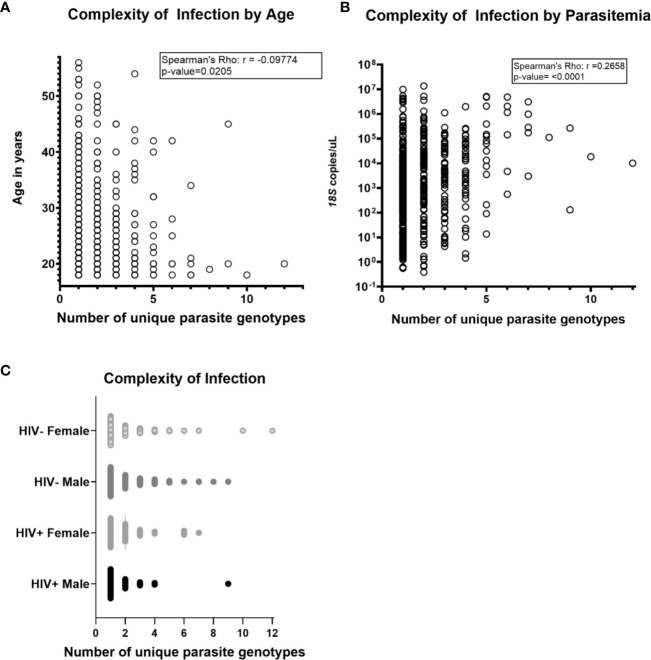
Complexity of *Plasmodium falciparum* infection (COI) was not associated with HIV-1 status, gender, age and parasitemia. COI is defined as the number of unique parasite genotypes as determined by encoded sequences for AMA1 d1, AMA1 d2, and CSP. Parasitemia was determined by *Plasmodium* genus-specific *18S ribosomal RNA* gene qPCR and HIV-1 status was determined by rapid diagnostic test (C. Kifude, pers. comm.). **(A)** Complexity of infection was not strongly correlated with age of volunteers by Spearman’s Rho nonparametric test for correlation (r=-0.09774, p-value=0.0205). **(B)** Complexity of infection was not strongly correlated with parasitemia (r=0.2658, p-value < 0.0001). **(C)** No significant differences in complexity of infection were detected in comparison by pairwise t-test between volunteers grouped by HIV-1 infection status and gender (n=563 volunteers; n=269 HIV-negative females, n=225 HIV-negative males; n=36 HIV+ females, n=33 HIV+ males). Two volunteers with inconclusive HIV-1 test results were excluded from this analysis.

Across the three groups of volunteers (COI=1, COI=2, COI>2), mean COIs based on any one locus (AMA1 d1, AMA1 d2, or CSP) were 2.34 and 2.26, and 2.02 respectively. However, across all volunteers with haplotype data for one, two, or three targets, mean COI was 1.50, 2.10, and 2.80, respectively, suggesting that our overall mean of 1.94 across all volunteers with any number of targets might be a conservative estimate. There were weak correlations between age and COI (**Figure 1A**) and between parasitemia and COI (**Figure 1B**), but there were no significant differences in COI by gender or HIV-1 status (**Figure 1C**). Collectively, these observations suggested that differences among patterns of DHFR and DHPS mutations within our volunteer population were not due to marked underlying differences in COI or parasitemia.

### Patterns of DHFR and DHPS Mutations in Our Study Volunteers Were Associated With HIV-1 Status in Age- and Gender-Specific Patterns

We first examined each encoded DHFR and DHPS mutations by HIV-1 status. The assumption of independence among these mutations is challenged by the coordinated appearance of DHFR and DHPS amino acid changes with increasing drug resistance (Plowe et al., 1997; Triglia et al., 1998; Mita et al., 2014; Cowell and Winzeler, 2019), but we chose to examine these mutations both independently and as haplotypes to detect any possible associations.

**Figure 2:**
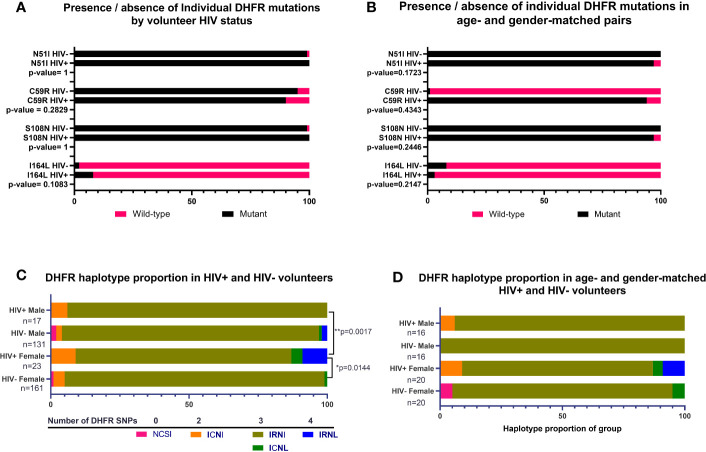
Presence of individual nonsynonymous mutations and proportions of DHFR haplotypes by HIV-1 status, or by age- and gender-matched pairs. **(A)** Individual mutations by HIV-1 status; n=292 nonsynonymous mutations identified from HIV-negative volunteers, n=40 nonsynonymous mutations identified from HIV-positive volunteers. **(B)** Individual mutations in age- and gender-matched, HIV-positive and HIV-negative volunteers; n=32 males, n=40 females. **(C)** Proportion of haplotypes in volunteers grouped by gender and HIV-1 status. The observed proportions were significantly different between HIV-positive versus HIV-negative females (p=0.0144) and between HIV-positive males versus HIV-positive females (p=0.0017) by Chi-square analysis. **(D)** Haplotypes of age- and gender-matched pairs grouped by HIV-1 status and gender. There were no significant differences in observed proportions of haplotypes when volunteers were matched by age and gender.

For DHFR, there were no significant associations between HIV-1 status and proportions of individual mutations across all volunteers or when volunteers were analyzed as age- and gender-matched pairs (**Figures 2A, B****)**. In contrast, we did detect significant differences in proportions of DHFR haplotypes among volunteers by HIV-1 status (**Figure 2C**). Specifically, DHFR haplotype proportions differed between HIV-positive and HIV-negative females (p=0.0144) and between HIV-positive males and HIV-positive females (p=0.0017). Here, 94% of HIV-negative females were positive for IRNI, while 78% of HIV-positive females were positive for IRNI. Further, 4% of HIV-positive females were positive for triple-mutant ICNL and 9% were positive for IRNL, the quadruple-mutant haplotype that is associated with high level SP resistance, while HIV-positive males lacked both ICNL and IRNL. However, these differences in proportions of DHFR haplotypes were no longer evident when volunteers were analyzed as age- and gender-matched pairs (**Figure 2D**), an outcome that may derive from the sample size reduction following volunteer pairing.

**Figure 3:**
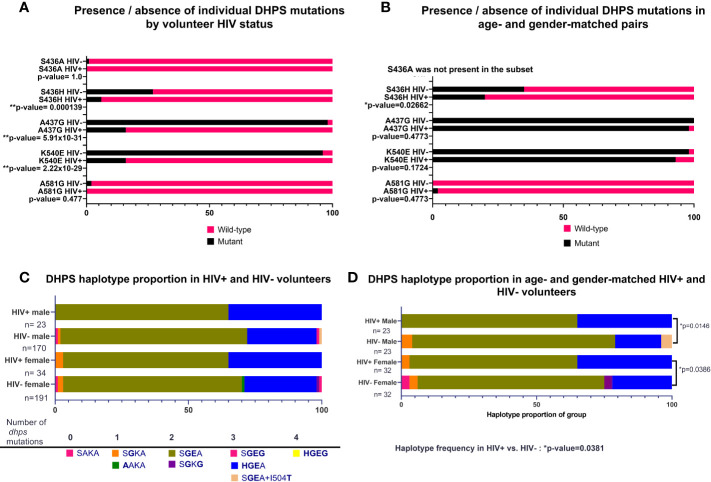
Presence of individual nonsynonymous mutations and proportions of DHPS haplotypes by HIV-1 status, or by age- and gender-matched pairs. **(A)** Individual mutations by HIV-1 status; n=361 mutations identified in HIV-negative volunteers, n=57 nonsynonymous mutations identified in HIV-positive volunteers. **(B)** Individual mutations in age- and gender-matched, HIV-positive and HIV-negative volunteers (n=46 males, n=64 females). S436H was significantly more prevalent in HIV-negative volunteers (p-value=0.02662). **(C)** Proportion of haplotypes in volunteers grouped by gender and HIV-1 status. **(D)** Haplotypes of age- and gender-matched pairs grouped by HIV-1 status. Significant differences were detected for HIV-positive males versus HIV-negative males (p=0.0146) and for HIV-positive females versus HIV-negative females (p=0.0386) by Chi-square analysis. Haplotype proportions were also significantly different between HIV-positive males and females (n=55) versus HIV-negative males and females (n=55, p-value=0.0381).

As for DHFR, the prevalences of individual DHPS mutations (K540E, A437G, S436H) were not significantly different by HIV-1 status in our volunteers (**Figure 3A**). When these data were re-analyzed following age- and gender-matching, a single mutation (S436H) was significantly different by HIV-1 status among age- and gender-matched pairs (p=0.02662; **Figure 3B**). When DHPS mutations were analyzed as proportions of haplotype frequencies, there were no significant differences among HIV-positive and HIV-negative volunteers (**Figure 3C**). Intriguingly, however, and in contrast to findings with DHFR, age- and gender-matching of our volunteers revealed significant differences in DHPS haplotypes by HIV-1 status (**Figure 3D**). Specifically, proportions of DHPS haplotypes differed between HIV-positive and HIV-negative males (p=0.0146) and between HIV-positive and HIV-negative females (p=0.0386; **Figure 3D**). While this finding is clearly independent of that for DHFR haplotypes (**Figure 2D**) where significant differences were lost following age- and gender-matching, our DHPS haplotype data indicate the potential to identify significant differences despite reduced statistical power associated with a smaller number of paired samples.

### Parasite Diversity Was Limited in Our Study Volunteers

**Figure 4:**
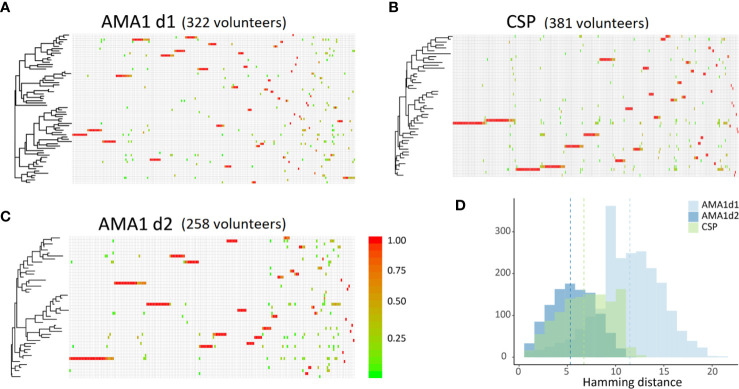
Sequence similarity among infecting strains of *P. falciparum* in our study volunteers. **(A–C)** Neighbor-joining phylogenies of the three loci used to determine COI. Haplotypes are given across rows, each column is an individual volunteer. Red indicates a single haplotype in a given volunteer, green indicates four or more haplotypes in a single volunteer. **(D)** Histogram of pairwise differences among haplotypes of *ama1 d1*, *ama1 d2*, and *csp*. Averages are indicated with dashed vertical lines. There are on average 5.3 nucleotide changes separating haplotypes of *ama1 d2* and 6.7 nucleotide changes separating haplotypes of *csp*. *ama1 d1* seems to suggest the strongest evolutionary potential with 10–15 nucleotides separating most unique haplotypes.

All three polymorphic loci for COI estimation performed equitably, returning sequence data for 322, 258, and 381 volunteers for AMA1 d1, AMA1 d2, and CSP (**Table 2**) which reduced to 131, 90, and 115 unique encoded haplotypes for AMA1 d1, AMA1 d2, and CSP, respectively. Using the haplotype sequences from SeekDeep we calculated pairwise differences, constructed Neighbor Joining phylogenies, and plotted within-host parasite composition for each volunteer (**Figure 4**). Slightly higher pairwise differences were observed for AMA1 d1 sequences than for AMA1 d2 and CSP. The average numbers of nucleotide differences between any two haplotypes of AMA1 d1, AMA1 d2, and CSP were 11.5, 5.3, and 6.7, respectively. Higher diversity in domain 1 of AMA1 has been observed previously and attributed to stronger balancing selection (Barry et al., 2009; Arnott et al., 2014; Zhu et al., 2016). Overall, the infecting parasites appeared to be divided into two clades containing most haplotypes (**Figure 4**) but this observation may not reflect a true bifurcation of the parasite population since each of our three loci cannot be linked in hosts with COI>1. Although there were many unique genotypes for each amplicon, only a handful of haplotypes were present in a large proportion of the samples (**Figure 4**). For each of the three amplicons, about one third of the volunteers had high within-host parasite diversity, comprised of genetically distant haplotypes. In general, however, volunteers with common haplotypes had low within-host parasite diversity, with only one haplotype present.

### In Volunteer Blood Samples With COI=1, *P. falciparum* DHFR and DHPS Mutation Prevalences Predicted Moderate Drug Resistance

**Figure 5:**
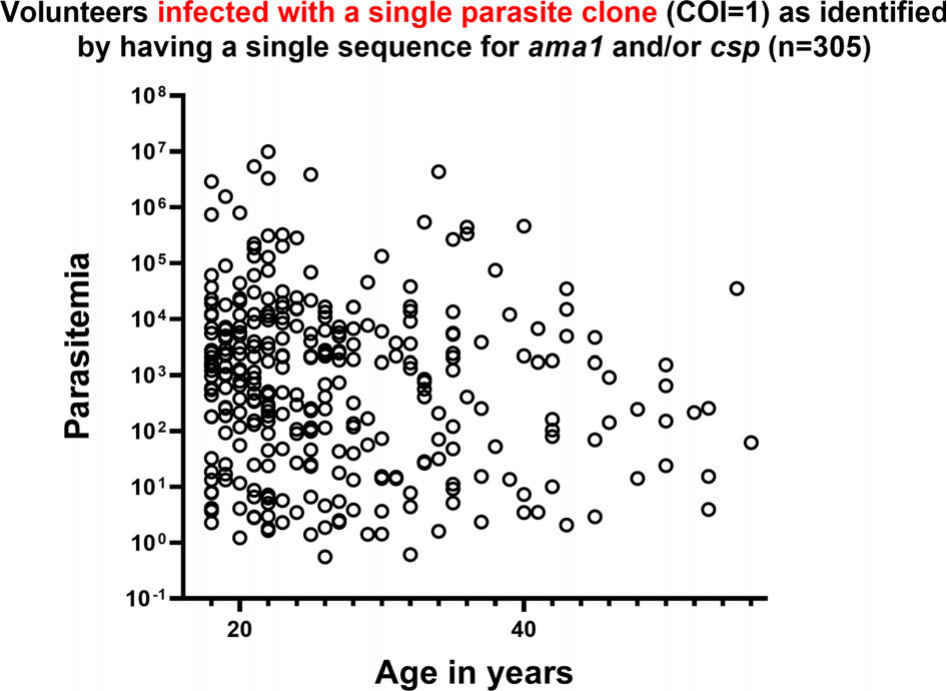
In volunteers infected with a single parasite clone (COI=1) of *P. falciparum* as identified by a single allele encoding AMA1 and/or CSP, parasitemia was highly variable across all ages. *P. falciparum 18S* copy number is represented as parasitemia on the y-axis in log scale. Age in years is represented on the x-axis.

Among the 305 individuals with COI=1, parasitemias were highly variable and showed no significant correlations with age (**Figure 5**) or with HIV-1 status (Chi-square, p=0.1,973). A total of 75 individuals with COI=1 had interpretable sequence data for both *dhfr* and *dhps*, allowing us to comment on haplotypes inclusive of both gene products or combined haplotypes (**Table 3**). Here, 18 volunteers were infected with the sextuple mutant IRNI-HGEA clone. One volunteer was infected with a clone with seven mutations across DHFR and DHPS with the haplotype IRNL-HGEA; the quadruple-mutant DHFR haplotype IRNL confers high-level resistance to pyrimethamine. One volunteer was infected with the quintuple mutant clone ICNI-HGEA and one volunteer was infected with the quintuple mutant ICNL-SGEA clone. A total of 54 volunteers were infected with the quintuple mutant clone IRNI-SGEA. A total of 5 of 75 volunteers were co-infected, four with the IRNI-SGEA clone (major haplotype for volunteers with COI=1) and one with the sextuple mutant IRNI-HGEA clone. By comparison, Juma et al. (Juma et al., 2019) noted that the IRNI-SGEA haplotype was significantly more prevalent in HIV-positive, co-infected volunteers randomized to stop Bactrim treatment (51.8%) relative to those who were HIV-positive and co-infected and remained on treatment after enrolling (6.3%). In our limited subset of volunteers newly diagnosed with HIV-1 with COI=1 and prior to administration of any HIV chemoprophylaxis, the presence of the quintuple mutant IRNI-SGEA was detectable in 4 of 5 of these individuals (80%).

**Table 3:** Dihydrofolate reductase and dihydropteroate synthase haplotypes in volunteers with COI=1*.

Combined haplotypes (DHFR-DHPS)mutant residues bolded	Number of mutations	Volunteers with this haplotype (%)
**I**C**N**I-**HGE**A	5	1 (1.3%)
**IRN**I-S**GE**A	5	54 (72%)
**I**C**NL**-S**GE**A	5	1 (1.3%)
**IRN**I-**HGE**A	6	18 (24%)
**IRNL-HGEA**	7	1 (1.3%)

*DHFR and DHPS haplotypes in volunteers determined as having a single circulating clone of P. falciparum by encoded sequences for AMA1 and CSP (n=75). Increasing resistance is shown for DHFR, DHPS and combined mutations based on published literature resistance (Plowe et al., 1997; Triglia et al., 1998; Mita et al., 2014; Cowell and Winzeler, 2019).

### Structural Analysis of I504T With A437G and K540E *P. falciparum* DHPS

The novel I504T mutation, confirmed in both PCR replicates, occurred with A437G and K540E in parasites from a single volunteer who was not co-infected with HIV-1. The parasitemia in this blood sample was ~3,387 *18S* copies/μl by qPCR with COI=2. The minor frequency clone (1%) included the I504T mutation along with A437G and K540E while the major clone included only A437G and K540E. While A437G and K540E are well known sulfa drug resistance mutations (Chitnumsub et al., 2019), the role of I504T is unknown, so we developed a homology model to predict substrate and drug binding interactions. The newly published *P. falciparum* DHPS model (Chitnumsub et al., 2019) included 13 crystal structures of different mutants in complex with sulfa drugs. **Supplemental Figure 2A** shows the superposition of our homology modeled structure of *P. falciparum* DHPS (violet) on the crystal structure (yellow) from Chitnumsub et al. (2019). The structural similarity is very high with an RMSD of 0.8 Å. Note that two inserts, insert-N3 (466-475) and insert-D7 (620-660), present in the crystal structure were not included in our homology modeled structure. **Supplemental Figure 2B** shows the superposition of endogenous ligands *p*ABA and DHPPP on the combined product pteroate (PTA, gray).

**Figure 6:**
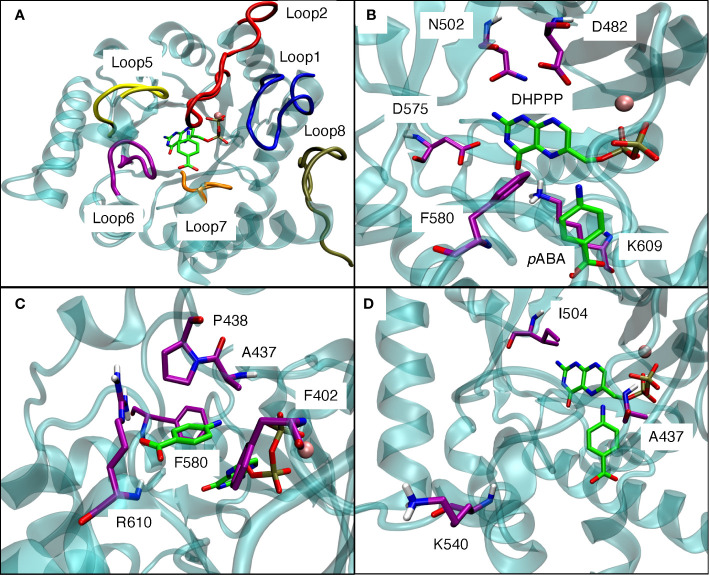
**(A)** Loop1, loop2, loop5, loop6, loop7 from the binding pocket for *p*ABA in modeled *P. falciparum* DHPS. **(B)** Binding interactions of DHPPP with modeled *P. falciparum* DHPS. **(C)** Binding interactions of *p*ABA with modeled *P. falciparum* DHPS. **(D)** Positions of three mutations around the binding site of endogenous ligands. Pink sphere is Mg^+2^ ion. Violet residues are involved in the binding sites. The endogenous ligands DHPPP and *p*ABA are represented in green.

**Figure 7:**
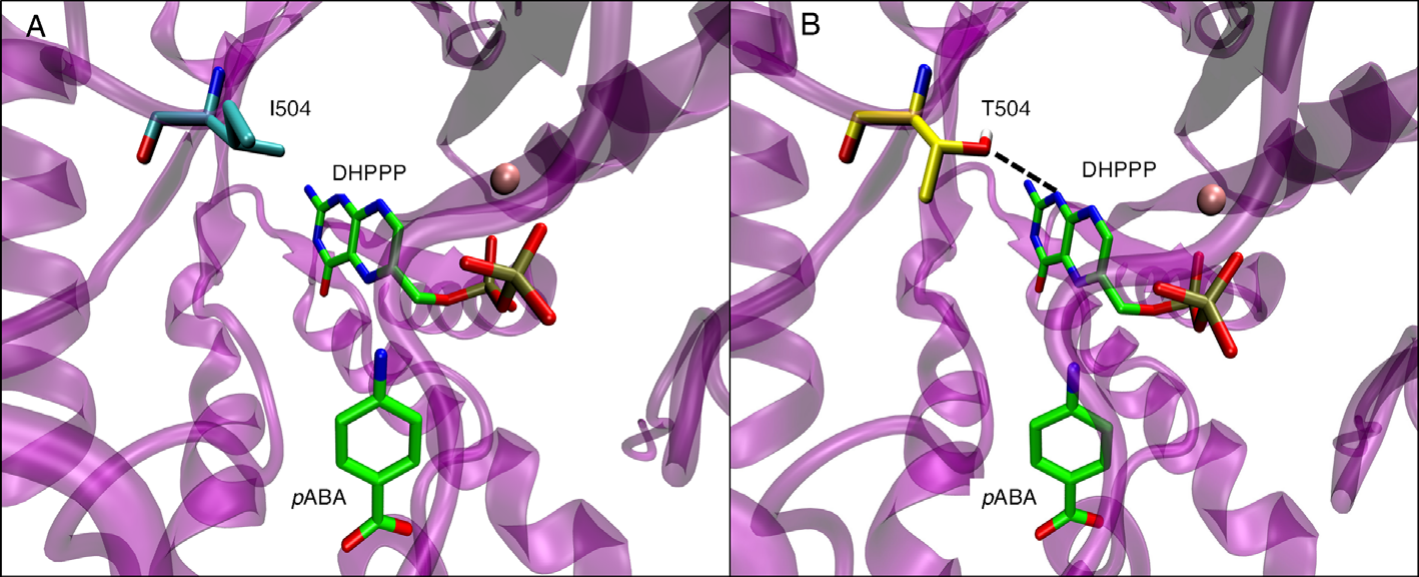
**(A)** Position of I504 within binding site of endogenous ligand DHPPP in wild type *P. falciparum* DHPS. I540 is shown in cyan. **(B)** H-bond interactions of T504 side chain with endogenous ligand DHPPP (dotted line) in mutated *P. falciparum* DHPS. T504 is shown in yellow. In both panels, pink sphere is Mg+2 ion and the endogenous ligands DHPPP, *p*ABA are represented in green.

The *p*ABA binding pocket in *P. falciparum* is formed by five loops: loop1, loop2, loop5, loop6, and loop7 (**Figure 6A**). **Figures 6B, C** show the binding interactions of the substrates DHPPP and *p*ABA with DHPS. The position of mutations A437, I504 and K540 are shown in **Figure 6D**. **Figure 7A** represents the location of I504 in the wild type structure of *P. falciparum* DHPS. I504 is present in the binding site of DHPPP and does not interact with DHPPP. **Figure 7B** shows the location of novel mutation I504T in the DHPPP binding site. The -OH group of the Thr sidechain forms an H-bond with DHPPP that increases the binding affinity of the mutant for DHPPP. This H-bond interaction was stable during the MD simulations and no other conformational changes were observed. We believe this predicted interaction is reasonable since I504T is located far away from the modeled loops and the inserts that were not included in our structure. Future studies are needed to determine whether I504T also affects enzymatic catalytic activity.

The well-known resistance mutation A437G is in the hydrophobic region of loop2 (A437, P438, F439, V440) in the binding site of *p*ABA. The Ala sidechain has a hydrophobic interaction with the benzene ring of *p*ABA. Mutation to Gly disrupts this hydrophobic interaction. In our MD simulations of the A437G mutant, loop2 becomes more flexible and different conformations were observed (**Supplemental Figure 3**). Similar conformational flexibility of the A437G mutant was discussed by Chitnumsub et al. (2019) in that this mutation reduced the binding affinity for PTA, but catalytic efficiency was increased. Our finding is consistent with other studies where Ala to Gly or Gly to Ala mutations significantly changed the conformational flexibility (Yan and Sun, 1997; Scott et al., 2007) and studies reporting that a Gly located adjacent to a Pro can alter conformations (Jacob et al., 1999).

**Figure 8:**
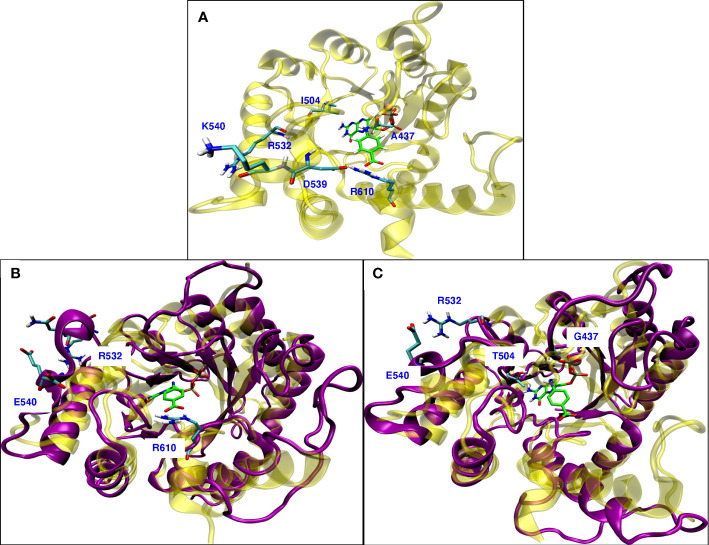
**(A)** Wild type *P. falciparum* DHPS structure: yellow with triple mutation sites and residues involved in rearrangement due to mutations shown in cyan. **(B)** Superposition of simulated modeled K540E *P. falciparum* DHPS structure (violet) on the crystal structure of K540E *P. falciparum* DHPS (yellow). New salt bridge formation between E540 and R532 due to conformational change while R610 maintains interactions with *p*ABA. **(C)** Superposition of simulated modeled triple mutant A437G/I504T/K540E *P. falciparum* DHPS structure (violet) on the crystal structure of wild type *P. falciparum* DHPS (yellow). New salt bridge formation is shown between E540 and R532 and all the loops around the binding sites have undergone conformational change but maintained the interactions with endogenous ligands. In all the panels, pink sphere is Mg+2 ion and the endogenous ligands DHPPP, *p*ABA are represented in green.

**Figure 8A** shows the wild type *P. falciparum* DHPS structure with triple mutation sites and the residues involved in the rearrangement due to conformational change observed because of mutations. Mutation K540E is located in the small helix turn after loop5, farther away from the binding sites for both the substrates (**Figure 8A**). Our MD simulations of the K540E mutant show that loop5 undergoes a conformational change forming a new stable H-bond with R532 (**Figure 8B**). The formation of this bond breaks the adjacent salt bridge between D539 and R610, but does not affect the H-bond interaction between R610 and *p*ABA. We believe this salt bridge breaking could be the molecular mechanism for the K540E drug resistance mutation. The breaking of the salt bridge between R610 and D539 was not observed in the newly published crystal structure (Chitnumsub et al., 2019), but their structure was a double mutant A437G/K540E in complex with pterin and p-hydroxy benzoic acid substrates which may explain the disagreement. Our MD simulations of the triple mutant A437G/I504T/K540E showed an additive effect of the conformational changes from both A437G and K540E (**Figure 8C**). With triple mutations, other than forming a new H-bond between E540 and R532, all loops involved in binding with endogenous ligands DHPPP and *p*ABA show the conformational changes.

## Discussion

In our cross-sectional study in western Kenya, HIV-1 co-infection in the absence of HIV chemotherapy was associated with altered proportions of individual mutations and haplotypes for DHFR and DHPS and these differences were variously dependent on age and gender both within the context of co-infection and relative to volunteers who were not co-infected (**Figures 2** and **3**). Based on weak correlations between age and COI and between parasitemia and COI and no differences in COI by gender and HIV-1 status, we inferred that these differences were not due to marked underlying differences in COI or parasitemia associated with gender, age or HIV-1 status. Because sequence data were not recovered for all three COI targets in all volunteers (**Table 2**), we cannot disregard the potential for underestimation of the number of clones per volunteer to impact our interpretations. Overall, the proportions of data captured (three targets per sample in each COI class) were 49% (444/915) for COI=1, 64% (242/381) for COI=2, and 69% (275/399) in COI>2. These observations suggest that with higher numbers of circulating clones in any volunteer, the probability of obtaining enough reads to reliably call an individual AMA1 or CSP allele in the population at a frequency of greater than 1% increases. In other words, there is potential for underestimation of true COI when minor allele frequencies are very small.

We identified in a single volunteer a novel DHPS mutation I504T, which we predict increases the affinity of DHPS for the endogenous substrate DHPPP. The I504T mutation occurred with A437G and K540E, two mutations that alter the conformation of the *p*ABA binding pocket and is predicted to function cooperatively with A437G and K540E to reduce drug binding. Our molecular modeling studies predicted conformational flexibility in loop2 with A437G mutation which is in agreement with a recently published structure (Chitnumsub et al., 2019). In contrast to A437G, our simulations of K540E showed a conformational change that suggests a possible drug resistance mechanism not observed in the crystal structure of double mutant A437G/K540E DHPS (Chitnumsub et al., 2019). Collectively, these observations indicate that antifolate resistance is continuing to evolve in Kenya and highlight the need to better understand the effects of associated mutations on both fitness and resistance in *P. falciparum*.

The effects of age on patterns of drug resistance are perhaps not surprising due to accumulated immunity, but the male-female differences are intriguing from the standpoint of the reported effects of gender on infection with a variety of pathogens (Garenne, 2015). While impacts of gender on risk of falciparum malaria have been studied (Bernin and Lotter, 2014), it remains difficult to determine the potential effects of a variety of factors on these patterns, including but not limited to gender-specific social behaviors (Diiro et al., 2016) and preferences for vector feeding on male and female human hosts (Okwa et al., 2011).

Clearly, the effects of HIV-1 status, prior to HIV chemotherapy, on patterns of drug resistance in *P. falciparum* are of concern, given that HIV co-infection reduces antimalarial efficacy due to a lack of supportive host immunity (Flateau et al., 2011). While co-infection with HIV-1 was not universally associated with increased diversity of DHFR and DHPS resistance haplotypes in our study, our data showed that larger proportions of co-infected volunteers were infected with parasite haplotypes with higher levels of drug resistance (**Figures 2C**, **3D**) despite relatively low parasite genetic diversity (**Figure 4**). These differences highlight the importance of resistance genotyping to develop the most effective therapeutic interventions for co-infection. Further, given that HIV-1 co-infected volunteers in our cohort were more likely to have mature falciparum gametocytes in circulation than volunteers with malaria alone (D. Stiffler, pers. comm.), we are currently evaluating whether co-infection not only increases transmission risk to exposed mosquitoes, but also transmission risk of drug-resistant genotypes of *P. falciparum*.

## Data Availability Statement

The datasets presented in this study are available from NCBI (https://www.ncbi.nlm.nih.gov/). Dataset: "Assembled population-level haplotypes of P. falciparum dhfr (PopSet: 1927947099: accession numbers MT886986 - MT886999), dhps (PopSet: 1927947063: accession numbers MT886968 - MT886985), csp (PopSet: 1927946859: accession numbers MT886866 - MT886967) and ama1 domain 1 (PopSet: 1927947308: accession numbers MT887090 - MT887221) and domain 2 (PopSet: 1927947128: accession numbers MT887000 - MT887089). Accession version(s) 1.

## Ethics Statement

The studies involving human participants were reviewed and approved by the Institutional Review Boards of the Uniformed Services University of the Health Sciences (USUHS# G18753), 4301 Jones Bridge Rd, Bethesda MD USA, Walter Reed Army Institute of Research IRB, 503 Robert Grant Ave, Silver Spring, MD USA(WRAIR #2033) and the Kenya Medical Research Institute (KEMRI, SSC protocol #2600; JO, Clinical Principle Investigator), Nairobi, Kenya. The patients/participants provided their written informed consent to participate in this study.

## Author Contributions

VS and SL conceived the project aims, obtained oversight approval, and planned the experiments. BT developed the methodology for high throughput library prep and devised its validation. CK compiled volunteer demographics, collected all samples and performed *Plasmodium* spp. qPCR assays with guidance and support from JW. ND and KR prepared all genomic DNA extracts from *Plasmodium*-positive volunteer samples. SG carried out all gDNA manipulations and next-generation sequencing library preparations. NH designed, optimized, and automated the primary data analysis pipeline. AM assisted with sequencing data curation and information technology support. JL performed parallel data analysis of raw sequencing data for external validation and conducted phylogenetic analyses of COI targets for diversity estimates. DP generated a homology structure of *P. falciparum* DHPS and conducted molecular dynamics simulations to investigate implications of novel I504T mutation alone and in conjunction with A437G and K540E mutations. JB designed primers for polymorphic target loci, contributed to the conceptualization of this work, and supported the use of computational resources for data analysis. BT, SL, DP, and JL wrote the manuscript. All authors provided timely and critical feedback for these studies which helped shape the research, data analyses, and manuscript preparation. All authors contributed to the article and approved the submitted version.

## Funding

This work was supported by NIH NIAID R01 AI104423 (VS, SL), NIH NIGMS P20 GM104420 (JL, DP) and the University of Idaho (SL). Data collection and analyses performed by the IBEST Genomics Resources Core at the University of Idaho were supported in part by NIH NIGMS P30 GM103324.

## Conflict of Interest

The authors declare that the research was conducted in the absence of any commercial or financial relationships that could be construed as a potential conflict of interest.

## Supplementary Material

Supplemental Table 1 Primers used for the generation of sequencing library templates.

Supplemental Figure 1 Raw reads were analyzed using SeekDeep Targeted Amplicon Analysis using default parameters. To confirm these results, raw reads were aligned using bwa-mem and SNPs were called using VCFtools from the GATK suite using default parameters proposed by both programs. The codon position of each SNP is printed along the x-axis. The y-axis (count) is the number of volunteers with parasites harboring mutations encoding those SNPs. SeekDeep data were used with aggregated counts for S436A and S436H. VCFtools called slightly fewer DHPS mutations compared with SeekDeep, while SeekDeep called slightly fewer DHFR mutations than VCFtools. SeekDeep identified the novel I504T encoded DHPS mutation, but this mutation was not called by VCFtools due to its rarity in the population.

Supplemental Figure 2 **(A)** Superposition of modeled structured of *P. falciparum* DHPS (violet) on the crystal structure of *P. falciparum* DHPS (yellow, 13). Co-crystalized ligand 1,3,5-Triaza-7-phosphaadamantane (PTA) is represented as grey carbon atoms and *p*ABA and DHPPP are represented as green carbon atoms. **(B)** Superposition of endogenous ligands within binding sites of *P. falciparum* DHPS. PTA is represented as grey carbon atoms and *p*ABA and DHPPP are represented as green carbon atoms. Pink sphere is Mg^+2^ ion.

Supplemental Figure 3 Superposition of simulated modeled A437G *P. falciparum* DHPS structure (violet) on the crystal structure of A437G *P. falciparum* DHPS (yellow). Loop2 is flexible in MD simulations.

## References

[B1] Abraham M. J. Murtola T. Schulz R. Páll S. Smith J. C. Hess B. (2015). GROMACS: High performance molecular simulations through multi-level parallelism from laptops to supercomputers. SoftwareX 1–2, 19–25. 10.1016/j.softx.2015.06.001

[B2] Arnott A. Wapling J. Mueller I. Ramsland P. A. Siba P. M. Reeder J. C. (2014). Distinct patterns of diversity, population structure and evolution in the AMA1 genes of sympatric *Plasmodium falciparum* and *Plasmodium vivax* populations of Papua New Guinea from an area of similarly high transmission. Malaria J. 13, 233. 10.1186/1475-2875-13-233 PMC4085730 24930015

[B3] Barry A. E. Schultz L. Buckee C. O. Reeder J. C. (2009). Contrasting population structures of the genes encoding ten leading vaccine-candidate antigens of the human malaria parasite, *Plasmodium falciparum*. PLoS One 4, e8497 (1:11). 10.1371/journal.pone.0008497 20041125 PMC2795866

[B4] Bernin H. Lotter H. (2014). Sex bias in the outcome of human tropical infectious diseases: influence of steroid hormones. J. Infect. Dis. 209, S107–S113. 10.1093/infdis/jit610 24966190

[B5] Caporaso J. G. Kuczynski J. Stombaugh J. Bittinger K. Bushman F. D. Costello E. K. (2010). QIIME allows analysis of high-throughput community sequencing data. Nat. Methods 7, 335–336. 10.1038/nmeth.f.303 20383131 PMC3156573

[B6] Chen S. Zhou Y. Chen Y. Gu J. (2018). fastp: an ultra-fast all-in-one FASTQ preprocessor. Bioinformatics 34, i884–i890. 10.1093/bioinformatics/bty560 30423086 PMC6129281

[B7] Chitnumsub P. Jaruwat A. Talawanich Y. Noytanom K. Liwnaree B. Poen S. The structure of *Plasmodium falciparum* hydroxymethyldihydropterin pyrophosphokinase-dihydropteroate synthase reveals the basis of sulfa resistance. FEBS J. 287, 3273–3297. 10.1111/febs.15196 31883412

[B8] Cowell A. N. Winzeler E. A. (2019). The genomic architecture of antimalarial drug resistance. Brief Funct. Genomics 18, 314–328. 10.1093/bfgp/elz008 31119263 PMC6859814

[B9] Danecek P. Auton A. Abecasis G. Albers C. A. Banks E. DePristo M. A. (2011). The variant call format and VCFtools. Bioinformatics 27, 2156–2158. 10.1093/bioinformatics/btr330 21653522 PMC3137218

[B10] de Roode J. C. Culleton R. Bell A. S. Read A. F. (2004). Competitive release of drug resistance following drug treatment of mixed *Plasmodium chabaudi* infections. Malaria J. 3, 33. 10.1186/1475-2875-3-33 PMC517944 15367331

[B11] Diiro G. M. Affognon H. D. Muriithi B. W. Wanja S. K. Mbogo C. Mutero C. (2016). The role of gender on malaria preventive behaviour among rural households in Kenya. Malaria J. 15, 14. 10.1186/s12936-015-1039-y PMC4704398 26738483

[B12] Dray S. Dufour A.-B. (2007). The ade4 Package: Implementing the Duality Diagram for Ecologists. J. Stat. Soft. 22, 1–14. 10.18637/jss.v022.i04

[B13] Duraisingh M. T. Curtis J. Warhurst D. C. (1998). *Plasmodium falciparum*: Detection of polymorphisms in the dihydrofolate reductase and dihydropteroate synthetase genes by PCR and restriction digestion. Exp. Parasitol. 89, 1–8. 10.1006/expr.1998.4274 9603482

[B14] Flateau C. Le Loup G. Pialoux G. (2011). Consequences of HIV infection on malaria and therapeutic implications: a systematic review. Lancet Infect. Dis. 11, 541–556. 10.1016/S1473-3099(11)70031-7 21700241

[B15] Garenne M. (2015). Demographic evidence of sex differences in vulnerability to infectious diseases: Table 1. J. Infect. Dis. 211, 331–332. 10.1093/infdis/jiu448 25114159 PMC4279781

[B16] Hathaway N. J. Parobek C. M. Juliano J. J. Bailey J. A. (2018). SeekDeep: single-base resolution de novo clustering for amplicon deep sequencing. Nucleic Acids Res. 46, e21 (1:13). 10.1093/nar/gkx1201 29202193 PMC5829576

[B17] Huang J. MacKerell A. D. (2013). CHARMM36 all-atom additive protein force field: Validation based on comparison to NMR data. J. Comput. Chem. 34, 2135–2145. 10.1002/jcc.23354 23832629 PMC3800559

[B18] Huang Y. Niu B. Gao Y. Fu L. Li W. (2010). CD-HIT Suite: a web server for clustering and comparing biological sequences. Bioinformatics 26, 680–682. 10.1093/bioinformatics/btq003 20053844 PMC2828112

[B19] Iyer J. K. Milhous W. K. Cortese J. F. Kublin J. G. Plowe C. V. (2001). *Plasmodium falciparum* cross-resistance between trimethoprim and pyrimethamine. Lancet 358, 1066–1067. 10.1016/S0140-6736(01)06201-8 11589941

[B20] Jacob J. Duclohier H. Cafiso D. S. (1999). The role of proline and glycine in determining the backbone flexibility of a channel-forming peptide. Biophys. J. 76, 1367–1376. 10.1016/S0006-3495(99)77298-X 10049319 PMC1300115

[B21] Jacobson M. P. Friesner R. A. Xiang Z. Honig B. (2002). On the role of the crystal environment in determining protein side-chain conformations. J. Mol. Biol. 320, 597–608. 10.1016/S0022-2836(02)00470-9 12096912

[B22] Jacobson M. P. Pincus D. L. Rapp C. S. Day T. J. F. Honig B. Shaw D. E. (2004). A hierarchical approach to all-atom protein loop prediction. Proteins: Struc. Funct. Bioinf. 55, 351–367. 10.1002/prot.10613 15048827

[B23] Juma D. W. Muiruri P. Yuhas K. John-Stewart G. Ottichilo R. Waitumbi J. (2019). The prevalence and antifolate drug resistance profiles of *Plasmodium falciparum* in study participants randomized to discontinue or continue cotrimoxazole prophylaxis. PLoS Negl Trop. Dis. 13, e0007223. 10.1371/journal.pntd.0007223 30897090 PMC6445470

[B24] Kalyesubula I. Musoke-Mudido P. Marum L. Bagenda D. Aceng E. Ndugwa C. (1997). Effects of malaria infection in human immunodeficiency virus type 1-infected Ugandan children. Pediatr. Infect. Dis. J. 16, 876. 10.1097/00006454-199709000-00011 9306483

[B25] Li H. Durbin R. (2009). Fast and accurate short read alignment with Burrows-Wheeler transform. Bioinformatics 25, 1754–1760. 10.1093/bioinformatics/btp324 19451168 PMC2705234

[B26] Li H. Handsaker B. Wysoker A. Fennell T. Ruan J. Homer N. (2009). The Sequence Alignment/Map format and SAMtools. Bioinformatics 25, 2078–2079. 10.1093/bioinformatics/btp352 19505943 PMC2723002

[B27] Luckhart S. Torrevillas B. K. Garrison S. M. McKeeken A. J. Patel D. Van Leuven J. T. (2020). Data available from NCBI GenBank (https://www.ncbi.nlm.nih.gov/). Assembled population-level haplotypes of P. falciparum dhfr (PopSet: 1927947099: MT886986 - MT886999), dhps (PopSet: 1927947063: MT886968 - MT886985), csp (PopSet: 1927946859: MT886866 - MT886967) and ama1 domain 1 (PopSet: 1927947308: MT887090 - MT887221) and domain 2 (PopSet: 1927947128: MT887000 - MT887089). Accession version(s) 1. August 14, 2020.

[B28] Manickam Y. Karl H. Sharma A. (2018). *RCSB PDB homepage*, released 08-28-2018. Available from: https://www.rcsb.org/structure/5Z79.

[B29] McKenna A. Hanna M. Banks E. Sivachenko A. Cibulskis K. Kernytsky A. (2010). The Genome Analysis Toolkit: a map-reduce framework for analyzing next-generation DNA sequencing data. Genome Res. 20, 1297–1303. 10.1101/gr.107524.110 20644199 PMC2928508

[B30] Missinou M. A. Lell B. Kremsner P. G. (2003). Uncommon asymptomatic *Plasmodium falciparum* infections in Gabonese children. Clin. Infect. Dis. 36, 1198–1202. 10.1086/374555 12715318

[B31] Mita T. Ohashi J. Venkatesan M. Marma A. S. P. Nakamura M. Plowe C. V. (2014). Ordered accumulation of mutations conferring resistance to sulfadoxine-pyrimethamine in the *Plasmodium falciparum* parasite. J. Infect. Dis. 209, 130–139. 10.1093/infdis/jit415 23922363

[B32] Okwa O. O. Bello B. A. Olundegun S. A. (2011). Human host preference of *Anopheles* mosquitoes collected from students hostels in a Nigerian university. South Asian J. Exp. Biol. 1 (3), 141–146.

[B33] Paradis E. Schliep K. (2019). ape 5.0: an environment for modern phylogenetics and evolutionary analyses in R. Bioinformatics 35, 526–528. 10.1093/bioinformatics/bty633 30016406

[B34] Plowe C. V. Cortese J. F. Djimde A. Nwanyanwu O. C. Watkins W. M. Winstanley P. A. (1997). Mutations in *Plasmodium falciparum* dihydrofolate reductase and dihydropteroate synthase and epidemiologic patterns of pyrimethamine-sulfadoxine use and resistance. J. Infect. Dis. 176, 1590–1596. 10.1086/514159 9395372

[B35] Rutto E. K. Nyagol J. Oyugi J. Ndege S. Onyango N. Obala A. (2015). Effects of HIV-1 infection on malaria parasitemia in milo sub-location, western Kenya. BMC Res. Notes 8, 303. 10.1186/s13104-015-1270-1 26173396 PMC4501056

[B36] Schliep K. P. (2011). phangorn: phylogenetic analysis in R. Bioinformatics 27, 592–593. 10.1093/bioinformatics/btq706 21169378 PMC3035803

[B37] Schroedinger Release (2020). “Maestro,” in Schrödinger Release 2020-2: Maestro. (New York, NY: Schrödinger, LLC). Available at: https://www.schrodinger.com/maestro.

[B38] Scott K. A. Alonso D. O. V. Sato S. Fersht A. R. Daggett V. (2007). Conformational entropy of alanine versus glycine in protein denatured states. PNAS 104, 2661–2666. 10.1073/pnas.0611182104 17307875 PMC1815238

[B39] Settles M. (2019). msettles/dbcAmplicons. Available at: https://github.com/msettles/dbcAmplicons (Accessed December 29, 2019).

[B40] Sondén K. Doumbo S. Hammar U. Vafa Homann M. Ongoiba A. Traoré B. (2015). Asymptomatic multiclonal *Plasmodium falciparum* infections carried through the dry season predict protection against subsequent clinical malaria. J. Infect. Dis. 212, 608–616. 10.1093/infdis/jiv088 25712968 PMC4539894

[B41] Steketee R. W. Wirima J. J. Bloland P. B. Chilima B. Mermin J. H. Chitsulo L. (1996). Impairment of a pregnant woman’s acquired ability to limit *Plasmodium falciparum* by infection with human immunodeficiency virus type-1. Am. J. Trop. Med. Hyg 55, 42–49. 10.4269/ajtmh.1996.55.42 8702036

[B42] Tadesse F. G. Slater H. C. Chali W. Teelen K. Lanke K. Belachew M. (2018). The relative contribution of symptomatic and asymptomatic *Plasmodium vivax* and *Plasmodium falciparum* infections to the infectious reservoir in a low-endemic setting in Ethiopia. Clin. Infect. Dis. 66, 1883–1891. 10.1093/cid/cix1123 29304258

[B43] Taylor S. M. Parobek C. M. Aragam N. Ngasala B. E. Mårtensson A. Meshnick S. R. (2013). Pooled deep sequencing of *Plasmodium falciparum* isolates: an efficient and scalable tool to quantify prevailing malaria drug-resistance genotypes. J. Infect. Dis. 208, 1998–2006. 10.1093/infdis/jit392 23908494 PMC3836461

[B44] Triglia T. Wang P. Sims P. F. G. Hyde J. E. Cowman A. F. (1998). Allelic exchange at the endogenous genomic locus in Plasmodium falciparum proves the role of dihydropteroate synthase in sulfadoxine-resistant malaria. EMBO J. 17, 3807–3815. 10.1093/emboj/17.14.3807 9669998 PMC1170716

[B45] Tukwasibwe S. Mugenyi L. Mbogo G. W. Nankoberanyi S. Maiteki-Sebuguzi C. Joloba M. L. (2014). Differential prevalence of transporter polymorphisms in symptomatic and asymptomatic falciparum malaria infections in Uganda. J. Infect. Dis. 210, 154–157. 10.1093/infdis/jiu044 24446524 PMC4162000

[B46] Wargo A. R. de Roode J. C. Huijben S. Drew D. R. Read A. F. (2007). Transmission stage investment of malaria parasites in response to in-host competition. Proc. R. Soc. B 274, 2629–2638. 10.1098/rspb.2007.0873 PMC1975767 17711832

[B47] Whitworth J. Morgan D. Quigley M. Smith A. Mayanja B. Eotu H. (2000). Effect of HIV-1 and increasing immunosuppression on malaria parasitaemia and clinical episodes in adults in rural Uganda: a cohort study. Lancet 356, 1051–1056. 10.1016/S0140-6736(00)02727-6 11009139

[B48] Yan B. X. Sun Y. Q. (1997). Glycine residues provide flexibility for enzyme active sites. J. Biol. Chem. 272, 3190–3194. 10.1074/jbc.272.6.3190 9013553

[B49] Ye J. Coulouris G. Zaretskaya I. Cutcutache I. Rozen S. Madden T. L. (2012). Primer-BLAST: A tool to design target-specific primers for polymerase chain reaction. BMC Bioinf. 13:134. 10.1186/1471-2105-13-134 PMC3412702 22708584

[B50] Yogavel M. Nettleship J. E. Sharma A. Harlos K. Jamwal A. Chaturvedi R. (2018). Structure of 6-hydroxymethyl-7,8-dihydropterin pyrophosphokinase–dihydropteroate synthase from *Plasmodium vivax* sheds light on drug resistance. J. Biol. Chem. 293, 14962–14972. 10.1074/jbc.RA118.004558 30104413 PMC6166723

[B51] Yu G. Smith D. K. Zhu H. Guan Y. Lam T. T.-Y. (2017). ggtree: an r package for visualization and annotation of phylogenetic trees with their covariates and other associated data. Methods Ecol. Evol. 8, 28–36. 10.1111/2041-210X.12628

[B52] Zhu X. Zhao Z. Feng Y. Li P. Liu F. Liu J. (2016). Genetic diversity of the *Plasmodium falciparum* apical membrane antigen I gene in parasite population from the China–Myanmar border area. Infect Genet. Evol. 39, 155–162. 10.1016/j.meegid.2016.01.021 26825252 PMC5069070

[B53] Zoete V. Cuendet M. A. Grosdidier A. Michielin O. (2011). SwissParam: A fast force field generation tool for small organic molecules. J. Comput. Chem. 32, 2359–2368. 10.1002/jcc.21816 21541964

