# Supplementary material for: Plasmodium falciparum DHFR and DHPS Mutations Are Associated With HIV-1 Co-Infection and a Novel DHPS Mutation I504T Is Identified in Western Kenya
Source: Front Cell Infect Microbiol. 2020 Nov 26;10:600112. doi: 10.3389/fcimb.2020.600112 (PMC7725689; doi:10.3389/fcimb.2020.600112)
Supplement: Supplemental Table 1 — Primers used for the generation of sequencing library templates. [file Table_1.docx]

**Supplemental Table 1:** Primers for initial PCR amplification and adapter extension reaction

| **Target** | **Primer names** | **Sequence (5’ 🡪 3’)** | **Amplicon size (bp)** | **Reference*** |
| --- | --- | --- | --- | --- |
| **Primary PCR** |  |  |  |  |
| *Pf* dihydrofolate reductase (*dhfr*) | *Pf*DHFR_Out_F | TCCTTTTTATGATGGAACAAG | 653 | Taylor et al. 2013 |
|  | *Pf*DHFR_Out_R | AGTATATACATCGCTAACAGA |  |  |
| *Pf* dihydro-pteroate synthase (*dhps*) | *Pf*DHPS_Out_F | AACCTAAACGTGCTGTTCAA | 711 | Duraisingh et al. 1998 |
|  | *Pf*DHPS_Out_R | AATTGTGTGATTTGTCCACAA |  |  |
| *Pf* apical membrane antigen 1, domain 1 (*ama1-*d1) | *Pf*AMA1d1_tspF | AGATTTAGGAGAAGATGCTGAAGT | 450 | This study |
|  | *Pf*AMA1d1_tspR | GCTGGTCTAAAACAAAACATGCTG |  |  |
| *Pf* apical membrane antigen 1, domain 2 (*ama1*-d2) | *Pf*AMA1d2_tspF | TCGGATTATGGGTCGATGGA | 446 | This study |
|  | *Pf*AMA1d2_tspR | TCGTTTTGATTCTCTTTCGATTTCT |  |  |
| *Pf c*ircumsporo-zoite protein encoding gene (*csp*) | *Pf*CSP_tspF | ATTGTACAACTCAAACTAAGATGTGT | 420 | This study |
|  | *Pf*CSP_tspR | GCAAATCCTAATAAAAACAATCAAGGT |  |  |
| **Nested-Adapter PCR** |  |  |  |  |
| *dhfr* | *Pf*DHFR_adF | ACACTGACGACATGGTTCTACACATTTAGAGGTCTAGGAAATAAAGG | 546 | This study |
|  | *Pf*DHFR_adR | TACGGTAGCAGAGACTTGGTCTTGATAAACAACGGAACCTCC |  |  |
| *dhps* | *Pf*DHPS_adF | ACACTGACGACATGGTTCTACAGCTAGTGTTATAGATATAGGTGG | 457 | This study |
|  | *Pf*DHPS_adR | TACGGTAGCAGAGACTTGGTCTGAGTTTAATAGATTGATCATGTTTC |  |  |
| *ama1* | *Pf*AMA1d1_adF | ACACTGACGACATGGTTCTACAAGATTTAGGAGAAGATGCTGAAGT | 501 | This study |
|  | *Pf*AMA1d1_adR | TACGGTAGCAGAGACTTGGTCTGCTGGTCTAAAACAAAACATGCTG |  |  |
| *ama1* | *Pf*AMA1d2_adF | ACACTGACGACATGGTTCTACATCGGATTATGGGTCGATGGA | 497 | This study |
|  | *Pf*AMA1d2_adR | TACGGTAGCAGAGACTTGGTCTTCGTTTTGATTCTCTTTCGATTTCT |  |  |
| *csp* | *Pf*CSP_adF: | ACACTGACGACATGGTTCTACAATTGTACAACTCAAACTAAGATGTGT | 471 | This study |
|  | *Pf*CSP_adR: | TACGGTAGCAGAGACTTGGTCTGCAAATCCTAATAAAAACAATCAAGGT |  |  |

*Unless otherwise indicated, primer sequences were designed by our group using NCBI Primer Blast (Ye et al. 2012)
