# Supplementary figures and images for: Plasmodium falciparum DHFR and DHPS Mutations Are Associated With HIV-1 Co-Infection and a Novel DHPS Mutation I504T Is Identified in Western Kenya
Source: Front Cell Infect Microbiol. 2020 Nov 26;10:600112. doi: 10.3389/fcimb.2020.600112 (PMC7725689; doi:10.3389/fcimb.2020.600112)

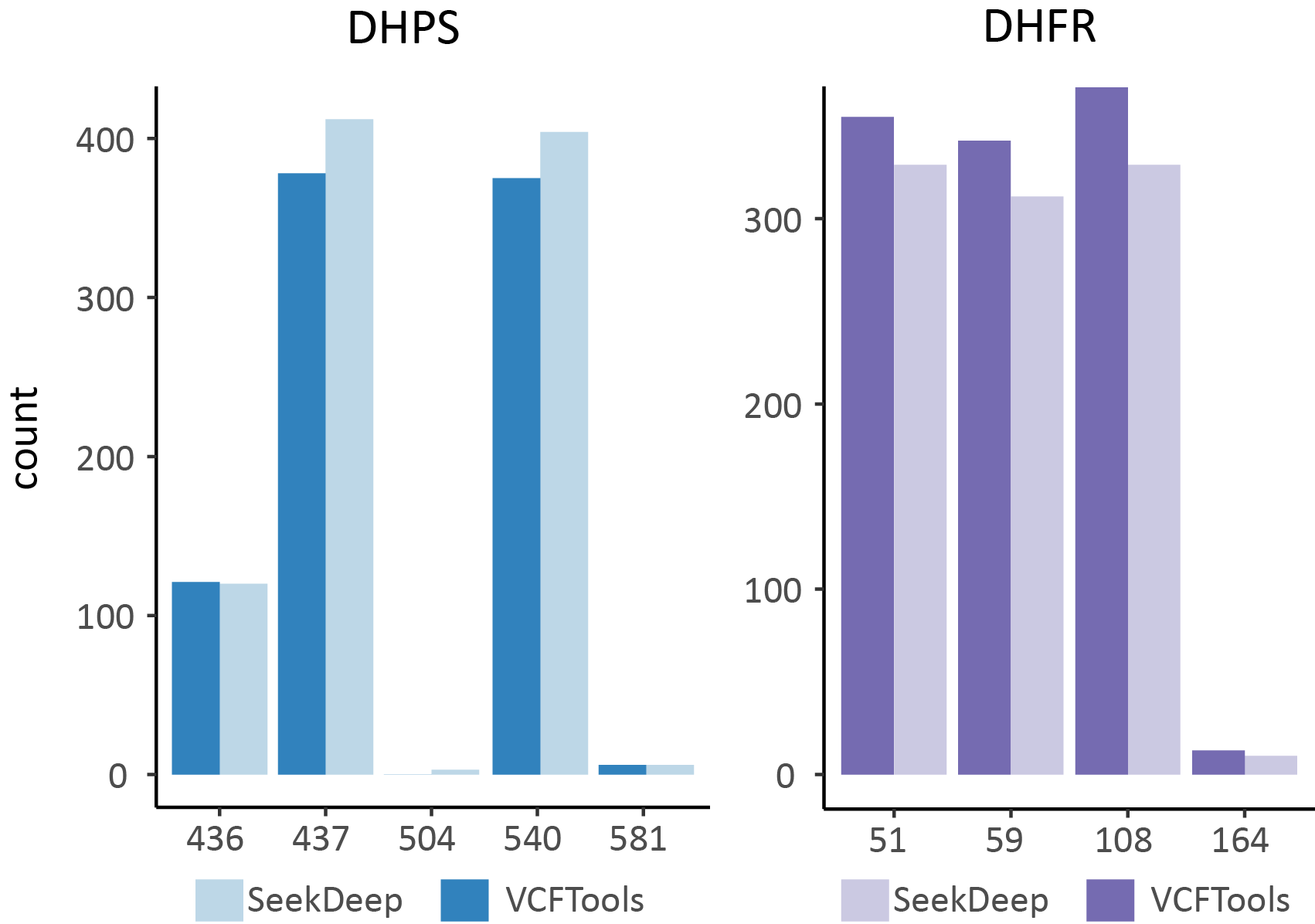

Supplement: Supplemental Figure 1 — Raw reads were analyzed using SeekDeep Targeted Amplicon Analysis using default parameters. To confirm these results, raw reads were aligned using bwa-mem and SNPs were called using VCFtools from the GATK suite using default parameters proposed by both programs. The codon position of each SNP is printed along the x-axis. The y-axis (count) is the number of volunteers with parasites harboring mutations encoding those SNPs. SeekDeep data were used with aggregated counts for S436A and S436H. VCFtools called slightly fewer DHPS mutations compared with SeekDeep, while SeekDeep called slightly fewer DHFR mutations than VCFtools. SeekDeep identified the novel I504T encoded DHPS mutation, but this mutation was not called by VCFtools due to its rarity in the population. [file Image_1.tif]

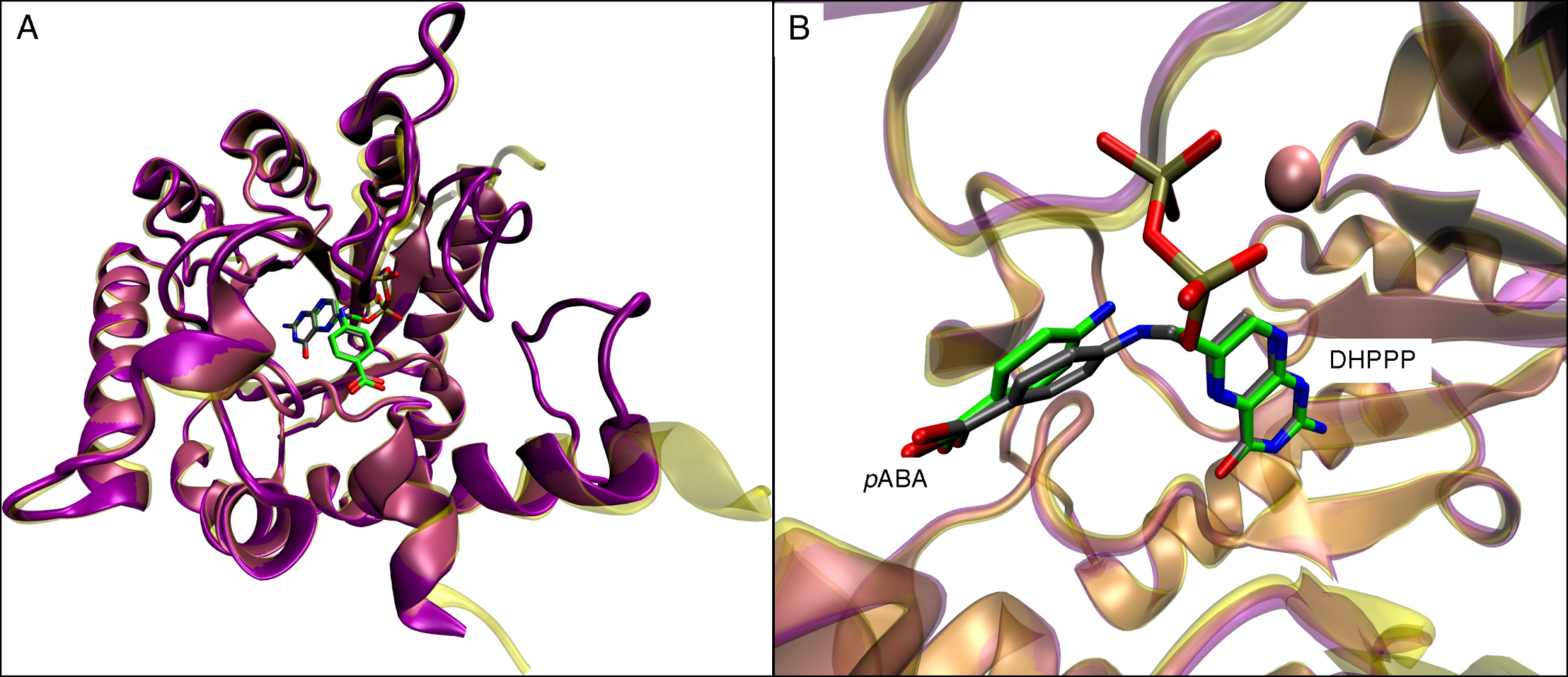

Supplement: Supplemental Figure 2 — (A) Superposition of modeled structured of P. falciparum DHPS (violet) on the crystal structure of P. falciparum DHPS (yellow, 13). Co-crystalized ligand 1,3,5-Triaza-7-phosphaadamantane (PTA) is represented as grey carbon atoms and pABA and DHPPP are represented as green carbon atoms. (B) Superposition of endogenous ligands within binding sites of P. falciparum DHPS. PTA is represented as grey carbon atoms and pABA and DHPPP are represented as green carbon atoms. Pink sphere is Mg+2 ion. [file Image_2.tif]

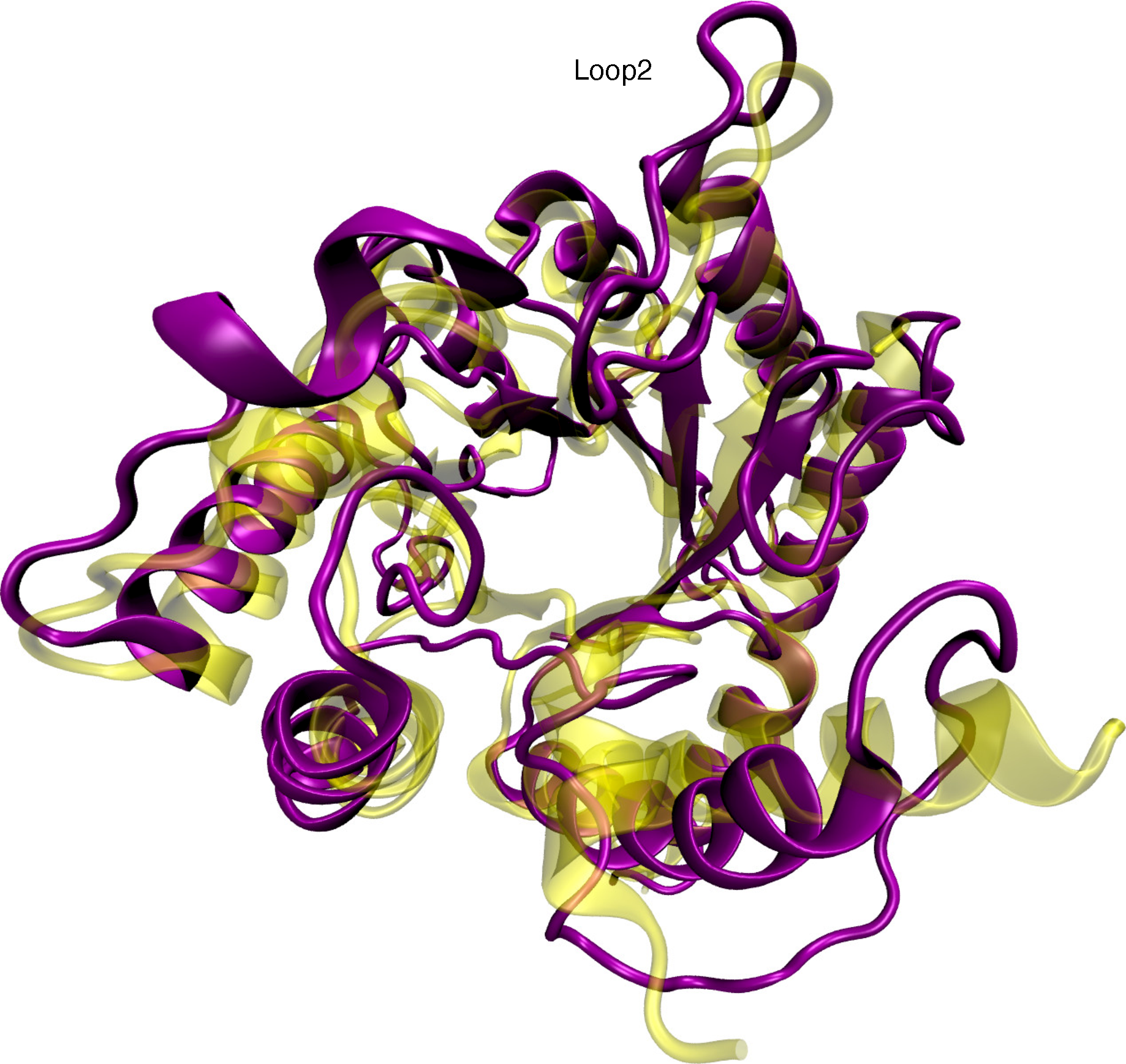

Supplement: Supplemental Figure 3 — Superposition of simulated modeled A437G P. falciparum DHPS structure (violet) on the crystal structure of A437G P. falciparum DHPS (yellow). Loop2 is flexible in MD simulations. [file Image_3.tif]
